# Targeting gut microbiota: a potential therapeutic approach for tumor microenvironment in glioma

**DOI:** 10.3389/fneur.2025.1549465

**Published:** 2025-03-21

**Authors:** Fan Qi, Kaiqiang Meng, Xiaoping Zhao, Jing Lv, Lan Huang, Xiaoxuan Fan, Zhaoqun Feng

**Affiliations:** ^1^College of Integrated Traditional and Western Medicine, Shaanxi University of Chinese Medicine, Shaanxi, China; ^2^College of Traditional Chinese Medicine, Shaanxi University of Chinese Medicine, Shaanxi, China; ^3^Neurosurgery Department of the Encephalopathy Hospital, Affiliated Hospital of Shaanxi University of Chinese Medicine, Shaanxi, China

**Keywords:** gut microbiota, microbiota-brain-gut axis, dysbiosis, tumor microenvironment, glioma

## Abstract

Glioma, being one of the malignant tumors with the highest mortality rate globally, has an unclear pathogenesis, and the existing treatment effects still have certain limitations. The tumor microenvironment (TME) plays an important role in the occurrence, development, and recurrence of glioma. As one of the important regulatory factors of TME, the gut microbiota can regulate the progression of glioma not only by interacting with the brain through the brain-gut axis but also by influencing the tumor immune microenvironment (TIME) and inflammatory microenvironment. Recent studies have identified the gut microbiota and TME as potential therapeutic targets for glioma. This paper aims to summarize the role of the gut microbiota in TME, the association between them and glioma, and the potential of developing new intervention measures by targeting the gut microbiota. Understanding the involvement process of the gut microbiota in glioma may pave the way for the development of effective treatment methods that can regulate TME and prevent disease progression.

## 1 Introduction

Glioma is the most common and highly malignant nervous system tumor. The continuous increase in its incidence and the challenges in treatment have made it one of the focuses of current medical research. This disease is characterized by high incidence, high recurrence rate, high mortality rate, and low cure rate. The incidence of glioma is approximately 5–8 cases per 100,000 people per year (1). Among them, glioblastoma (GBM) is the glioma type with the highest malignant grade. The prognosis of GBM patients is poor, with a median survival time of only about 14.6–16.7 months. Despite decades of research, the pathogenesis of glioma remains unclear. Moreover, after treatments such as surgery, radiotherapy, and chemotherapy, the survival time and quality of life of most patients with high-grade glioma cannot be guaranteed, and the prognosis is poor (2, 3). However, recent studies have revealed that the occurrence and development of glioma are usually caused by the combined action of multiple factors. Among them, the TME plays a crucial role in this process, providing a new approach for the intervention and treatment of glioma (4).

The TME is the functional and structural niche for tumor occurrence and progression. In the TME, tumor-associated immune cells, stromal cells and others are widely aggregated. The existence of these cells significantly enhances the proliferative ability of tumor cells, enabling them to grow and divide rapidly and which strengthens the migratory ability of tumor cells, prompting the spread of tumors to surrounding tissues and organs. Meanwhile, it also improves the immune escape ability of tumor cells, allowing them to evade the attacks of the body’s immune system. The TME will also affect the functional metabolism of tumor cells, change the ways of energy acquisition and material metabolism of tumor cells, thus providing favorable conditions for tumor growth (5, 6). In addition, the TME can induce the formation of an immunosuppressive microenvironment, inhibit the killing effect of the immune system on tumors, and then promote the development and metastasis of tumors (7). Existing studies have shown that the gut microbiota is an important regulatory factor of the TME, and gut microbiota dysbiosis may lead to TME remodeling and abnormal immune responses, thereby promoting tumor progression (8, 9).

**Figure 1:**
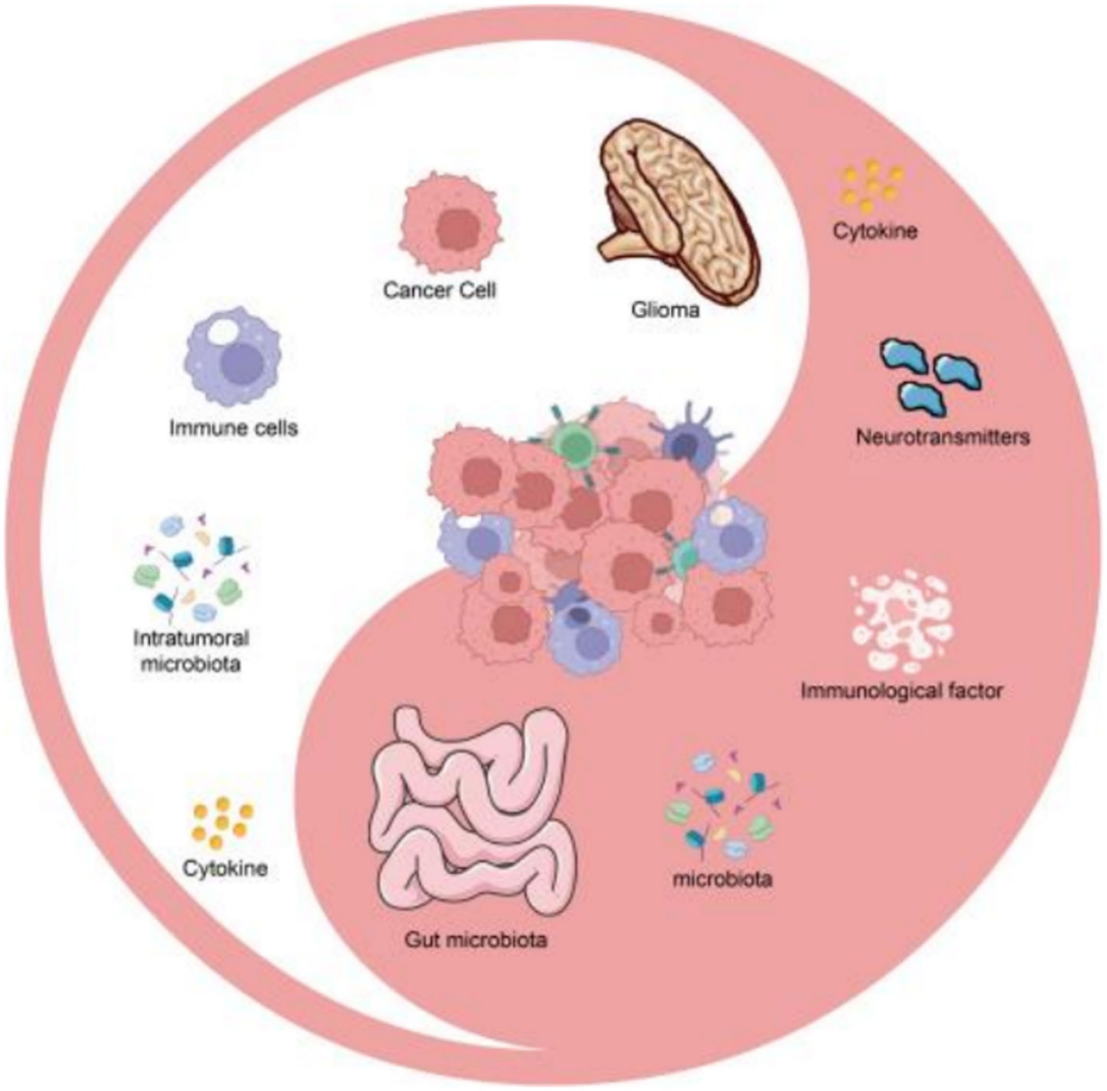
The key mechanisms for gut microbiota intervening in glioma by regulating the tumor microenvironment.

Targeted regulation of the gut microbiota holds significant prospects as a treatment approach for glioma. By restoring the balanced state of the gut microbiota and regulating the changing trend of the TME, it is possible to alleviate the symptoms of glioma and halt the progression of the tumor (10). In this paper, we aim to comprehensively summarize the role of the gut microbiota in regulating the TME and its association with glioma. We will explore the potential mechanisms of gut microbiota dysbiosis in the progression of glioma and discuss the potential of targeted regulation of the gut microbiota as a treatment strategy for glioma. Understanding the role of the gut microbiota in the progression of glioma may pave the way for the development of effective treatment measures that can regulate the TME and prevent the progression of the disease (Figure 1).

## 2 The interactive relationship between TME microecology and glioma

The concept of the TME evolved from the “seed-soil” theory, emphasizing that tumor development depends not only on the tumor cells themselves but also on their surrounding environment. The local environment around the tumor constitutes the necessary material basis for tumor growth, development, and metastasis (11, 12). The TME is a highly heterogeneous dynamic microecosystem (13). Besides tumor cells, it consists of various cell and non-cell components, such as immune cells like T cells, macrophages, and NK cells, stromal cells like fibroblasts and endothelial cells, and signal factors like cytokines, growth factors, and extracellular vesicles (14). In addition, the intratumoral microbiota is also an important part of the TME (15). The discovery of the intratumoral microbiota has greatly enriched the architecture of the TME microecosystem and has become one of the key factors in regulating the progression of tumor diseases. Studies have shown that the ability of glioma to metastasize and invade is affected not only by gene mutations but also closely related to the TME microecology in which it is located (16). Meanwhile, the high heterogeneity of glioma can lead to TME reprogramming, which in turn has an impact on the drug resistance of tumors and the prognosis of patients (17).

### 2.1 The influential effects of tumor-associated immune cells on glioma

In the TME, glioma cells, together with various types of immune cells, complex extracellular matrices, chemokines, and immune checkpoint molecules, jointly influence the construction of the TME (18). Over-activated tumor cells activate and mobilize immunosuppressive cells, promoting the development of the immune microenvironment toward an immunosuppressive state, thereby playing a role in resisting immune responses. The balanced state of the tumor immune system is closely related to whether the tumor progresses or not (19).

Studies have shown that the biological functions of tumor-associated immune cells are closely related to the prognosis and overall survival rate of glioma patients (20). In the glioma TME, tumor-associated macrophages (TAMs) accumulate in large numbers and exhibit significant phenotypic heterogeneity. They are mainly divided into M1 type with anti-tumor activity and M2 type that promotes tumor progression. Studies in recent years have shown that although M1-type TAMs can induce apoptosis of tumor cells by secreting reactive oxygen species (ROS) and pro-inflammatory cytokines (such as TNF-α, IL-12), they generally undergo phenotypic reprogramming in the glioma TME and ultimately polarize into the M2 type (21). This polarization switch is mainly driven by specific molecular signals in the TME. In particular, cytokines such as IL-4, IL-10, and TGF-β secreted by glioma cells, as well as chemokines such as CCL2 and CSF-1, jointly form a complex regulatory network that plays a crucial role in the polarization transition from the M1 type to the M2 type (22, 23). The M2-type TAMs release a variety of tumor-promoting cytokines and growth factors, inducing an anti-inflammatory response in tumor cells. This, in turn, enables tumor cells to successfully evade immune surveillance (24). Meanwhile, M2-type TAMs can also promote angiogenesis, matrix remodeling, and cellular drug resistance, directly or indirectly promoting the progression and metastasis of tumor cells (25). In addition, neutrophils also play an important role in glioma. In human brain glioma, the number of circulating and infiltrating neutrophils is correlated with the glioma grade. Neutrophil infiltration is related to the acquired resistance to anti-vascular endothelial growth factor therapy and can promote tumor growth and invasion. Research has found that neutrophils produce arginase I, inducing immune suppression and thereby promoting tumor growth. At the tumor site, neutrophils secrete elastase to assist glioma infiltration and simultaneously produce S100a4, which directly induces the proliferation of GBM cells (26). Dendritic cells (DCs) recognize and capture tumor cells, transmit antigens to activate the body’s immune response, and regulate the immune state in the TME (27). However, the brain is essentially deficient in DCs, and the immune efficacy of DCs in glioma cannot be fully manifested. T cells and natural killer (NK) cells, as key mediators in tumor immunotherapy, inhibit the growth and spread of glioma by monitoring and eliminating tumor cells, promoting the apoptosis or necrosis of tumor cells. However, due to the existence of the blood–brain barrier in the central nervous system, antigen presentation is difficult, the entry of T cells and antibodies is restricted, and the immune function is often limited, allowing glioma to grow and invade (28, 29). Regulatory T cells (Tregs) increase in glioma and can promote tumor growth by suppressing the activity of effector T cells and creating an immunosuppressive microenvironment (30). Meanwhile, various immunosuppressive molecules in the TME can reduce the activity of immune cells, inhibit immune responses, and further affect the state of the TME (31).

In conclusion, in the TME, glioma cells promote the invasion of tumor cells and evade the surveillance and attack of the immune system by interfering with the functions and interactions of TAMs, T cells, NK cells, and DCs (32, 33). It can be seen from this that reshaping the phenotypes of malignant cells in the TME and regulating the immunosuppressive microenvironment have great potential in reversing the progression of glioma, which is worthy of further research in the future.

### 2.2 The influential effects of tumor-associated stromal cells on glioma

The TME of glioma usually presents an acidic and hypoxic environment. Tumor-associated stromal cells, under continuous stimulation, can interact with tumor cells in a complex manner through multiple pathways, thereby promoting the growth, invasion, and metastasis of glioma (34, 35).

Tumor-associated fibroblasts (CAFs) can secrete growth factors such as hepatocyte growth factor (HGF), platelet-derived growth factor (PDGF), and transforming growth factor-β (TGF-β), which directly stimulate the proliferation and survival of glioma cells (36). CAFs can also secrete extracellular matrix (ECM) components such as collagen and fibronectin to provide support and nutrition for tumor cells, thereby promoting the growth and invasion of glioma (37). Studies have also found that CAFs can secrete immunosuppressive factors, such as prostaglandin E2 (PGE2) and indoleamine 2,3-dioxygenase (IDO), to inhibit the activity of immune cells, thus promoting the immune escape of tumors (38). The glioma TME contains abundant tumor microvessels, pericytes, and CAFs, which provide the necessary material and energy basis for the proliferation and invasion of glioma cells (39). Glioma cells rely on vascular supply of nutrients and oxygen to promote growth and achieve diffusion in brain tissue through vascular endothelial cells. Vascular endothelial cells are stimulated by pro-angiogenic factors such as vascular endothelial growth factor (VEGF) and basic fibroblast growth factor (bFGF) to promote their proliferation, migration, and formation of new blood vessels (40, 41). Meanwhile, endothelial cells provide a scaffold structure for new blood vessels and interact with surrounding cells to promote the differentiation and development of tumor neovascularization (42). In glioma, pericytes are located around the vascular wall and interact with endothelial cells to maintain vascular stability. A decrease in the number or abnormal function of pericytes may lead to an increase in vascular permeability, promoting the growth and transfer of glioma (43, 44). Studies have also found that astrocytes and microglia play an important role in the occurrence and development of glioma. They can secrete various cytokines and chemokines to regulate extracellular matrix remodeling, thereby promoting the growth and invasion of glioma (45). In addition, tumor-associated stromal cells can also interact with glioma cells to regulate the metabolism and signal transduction of tumor cells, promoting the growth and survival of tumors (46).

In conclusion, tumor-associated stromal cells play an important role in the occurrence and development of glioma. The adaptive signal pathways in the TME of glioma are widely activated. Tumor-associated stromal cells affect the metabolic activity and functions of mesenchymal cells and extracellular matrix directly or indirectly, generating more growth factors, pro-angiogenic factors, and immunosuppressive factors, etc. Consequently, a series of changes such as epithelial-mesenchymal transition, tumor stem cell differentiation, vasculogenic mimicry formation, and tumor cell immune escape occur.

### 2.3 The influential effects of tumor-associated cytokines on glioma

Tumor-associated cytokines influence the progression of glioma in various ways, mainly including promoting tumor growth and resisting the anti-tumor immune response.

In the TME of glioma, the cytokines that play a role in promoting glioma growth mainly include VEGF, PDGF, and TGF-β, etc. Glioma cells can secrete a large amount of VEGF, which stimulates the proliferation, migration, and formation of new blood vessels by endothelial cells. The abundant tumor blood vessels provide oxygen and nutrients for tumor cells, promoting tumor growth and invasion (47). PDGF promotes the growth, survival, and migration of glioma cells by activating its receptors. Meanwhile, PDGF can also induce the activation of tumor-associated fibroblasts, promoting the remodeling of the TME (48, 49). TGF-β has multiple biological functions. It can induce the epithelial-mesenchymal transition of glioma cells and the generation of tumor microvessels, enhancing the migration and invasion abilities of tumor cells (50). Meanwhile, TGF-β can also inhibit the activity of immune cells, promoting the immune escape of tumors (51).

In the TME, interferon-γ (IFN-γ), interleukin-2 (IL-2), and tumor necrosis factor-α (TNF-α) play roles in anti-tumor immunity. IFN-γ can promote the polarization of TAMs toward the M1 type, enhancing their anti-tumor activity. Meanwhile, IFN-γ can also upregulate the expression of major histocompatibility complex (MHC) molecules on the surface of tumor cells, enhancing the immunogenicity of tumor cells and facilitating the recognition and killing by T cells (52). IL-2 can promote the proliferation and activation of T cells and NK cells, enhancing the anti-tumor activity of immune cells and promoting the apoptosis of glioma cells (53). TNF-α has an anti-tumor effect. At low concentrations, TNF-α can activate immune cells and enhance the anti-tumor immune response. At high concentrations, TNF-α can directly kill tumor cells, but it will also cause severe inflammatory reactions and tissue damage (54, 55).

### 2.4 The influential effect of intratumoral microbiota on glioma

Intratumoral microbiota refers to the bacterial communities that exist within and around tumor tissues. As a new and integral component of the tumor system, it is an indispensable part of the TME (56). Studies have shown that the composition of intratumoral microbiota in tumors at different sites is not exactly the same, and the abundance and diversity of the microbiome are closely related to tumor occurrence, development, and metastasis, etc. (57).

After identifying and quantifying more than 1,500 samples of different types of tumor tissues and adjacent normal tissues by methods such as 16S ribosomal gene sequencing and quantitative PCR, the presence of bacterial DNA was detected in some tumor tissues that have no direct contact with the external environment, such as glioblastoma, breast tumors, and bone tumors (58). This opens up a new perspective for cancer research and is of great significance for comprehensively understanding the biological characteristics of tumors and developing new methods for tumor diagnosis and treatment. In glioma research, a 3D imaging protocol has been developed by combining tissue clearing, immunofluorescence labeling, optical section microscopy, and image processing techniques. This protocol can visualize and quantify the microbial communities within glioma tissues in a non-contaminating way. Moreover, it has been determined that bacterial DNA exists within glioma tissues, and in particular, the bacterial lipopolysaccharide (LPS) signal has been detected. They also found that the LPS signal shows an irregular shape and sparse distribution, mainly located near the nuclear membrane or in the intercellular space (59). Furthermore, mechanistic studies suggest that there is a characteristic disorder in the structure of the microbial community within glioma tissues. This is mainly manifested as a significant increase in the abundance of bacteria such as *Fusobacterium nucleatum*, Granulicatella, Enteromonas, Pasteurella, *Lactobacillus mucosae*, and Arthrobacter. Enrichment of bacterial RNA and LPS has also been detected within the tumor. Through further multi-omics integration analysis and experimental verification, it has been shown that genes related to the intratumoral microbiota are specifically enriched in the synaptic signaling pathway. By activating the neurotransmitter regulatory network, this significantly upregulates the expression levels of neuron-specific genes, thereby driving the malignant proliferation process of glioma cells (60). Although current research on the intratumoral microbiota of gliomas is still in its infancy, as a key component of the tumor microenvironment, its potential value as a novel diagnostic biomarker and a precise intervention target is beginning to emerge, making it worthy of in-depth study (Table 1).

**Table 1:** The impact of components in the tumor microenvironment on glioma.

Cell types	Cell type	Mechanism of action	Effect	References
Tumor-associated immune cells	M1 TAMs	Promote oxidation reactions and inflammatory responses, and facilitate the apoptosis of tumor cells	Anti-tumor	Ren et al. (21)
	M2 TAMs	Promote angiogenesis, matrix remodeling and drug resistance of cells	Tumor-promoting	Boutilier and Elsawa (22), Basak et al. (23), Yang et al. (24), Lin et al. (25)
	Neutrophils	Induce immune regulation	Tumor-promoting	Chang et al. (26)
	DC	Promote immune responses	Anti-tumor	Zhou et al. (27)
	T cells	Promote immune responses and facilitate the apoptosis of tumor cells	Anti-tumor	Liu et al. (28), Friedrich et al. (29)
	NK cells	Promote immune responses and facilitate the apoptosis of tumor cells	Anti-tumor	Liu et al. (28), Friedrich et al. (29)
	Tregs	Inhibit immune responses	Tumor-promoting	Lin et al. (30)
Tumor-associated stromal cells	Tumor-associated fibroblasts	Directly stimulate the proliferation of glioma cells and inhibit immune responses	Tumor-promoting	Galbo et al. (36), Jain et al. (37), Zhang et al. (38), Blonska et al. (39)
	Vascular endothelial cells	Facilitate the migration and invasion of tumor cells and promote angiogenesis	Tumor-promoting	Ahir et al. (40), Chryplewicz et al. (41), Xie et al. (42), Hoogstrate et al. (43), Cheng et al. (44)
	Astrocytes and microglia	Regulate extracellular matrix remodeling	Tumor-promoting	Blanco-Carmona et al. (45), Andersen et al. (46)
Tumor-associated cytokines	VEGF	Promote angiogenesis	Tumor-promoting	Miletic et al. (47)
	PDGF	Directly stimulate the proliferation of glioma cells	Tumor-promoting	Lv et al. (48), Kesari and Stiles (49)
	TGF-β	Promote epithelial-mesenchymal transition and angiogenesis, and inhibit immune responses	Tumor-promoting	Joseph et al. (50), Hou et al. (51)
	IFN-γ	Promote the polarization of TAMs to the M1 type and enhance immune responses	Anti-tumor	Pyonteck et al. (52)
	IL-2	Promote immune responses	Anti-tumor	Bommareddy et al. (53)
	TNF-α	Promote immune responses and inflammatory responses	Anti-tumor	Sanchez et al. (54), Lei et al. (55)

Overall, the imbalance of the immune system is a key driving factor for glioma cells to achieve immune escape. On the one hand, glioma cells suppress the function of the immune system and interact with inhibitory immune cells, stromal cells and various signaling factors to jointly construct an immunosuppressive TME. On the other hand, glioma cells have complex and frequent interactions with multiple components in the TME, creating a microenvironment conducive to tumor growth and metastasis, thereby accelerating the development process of glioma.

## 3 The influence pathways of gut microbiota on glioma

At present, it is believed that, in addition to directly invading glioma by disrupting the blood-brain barrier, gut microbiota mainly acts on glioma through the gut-brain axis, changes the gut-immune-brain communication, and forms a tumor-tolerant microenvironment in the central nervous system, thus promoting the development of glioma (61).

The gut-brain axis refers to a two-way information-communication system between the brain and the intestine and its microbiota (62). The gut-brain axis plays an important regulatory role in the human nervous system, digestive system and immune system. A complete gut-brain axis depends on healthy gut microbiota, an intact intestinal barrier and normal brain function (63). The inter–relationship between the brain and the intestine is mainly closely connected through a network formed by neuroendocrine pathways, metabolic pathways, immune pathways and the vagus nerve and other routes. They interact with substances such as hormones, neurotransmitters and short-chain fatty acids, constituting a two-way regulatory relationship of the gut-brain axis (64, 65).

**Figure 2:**
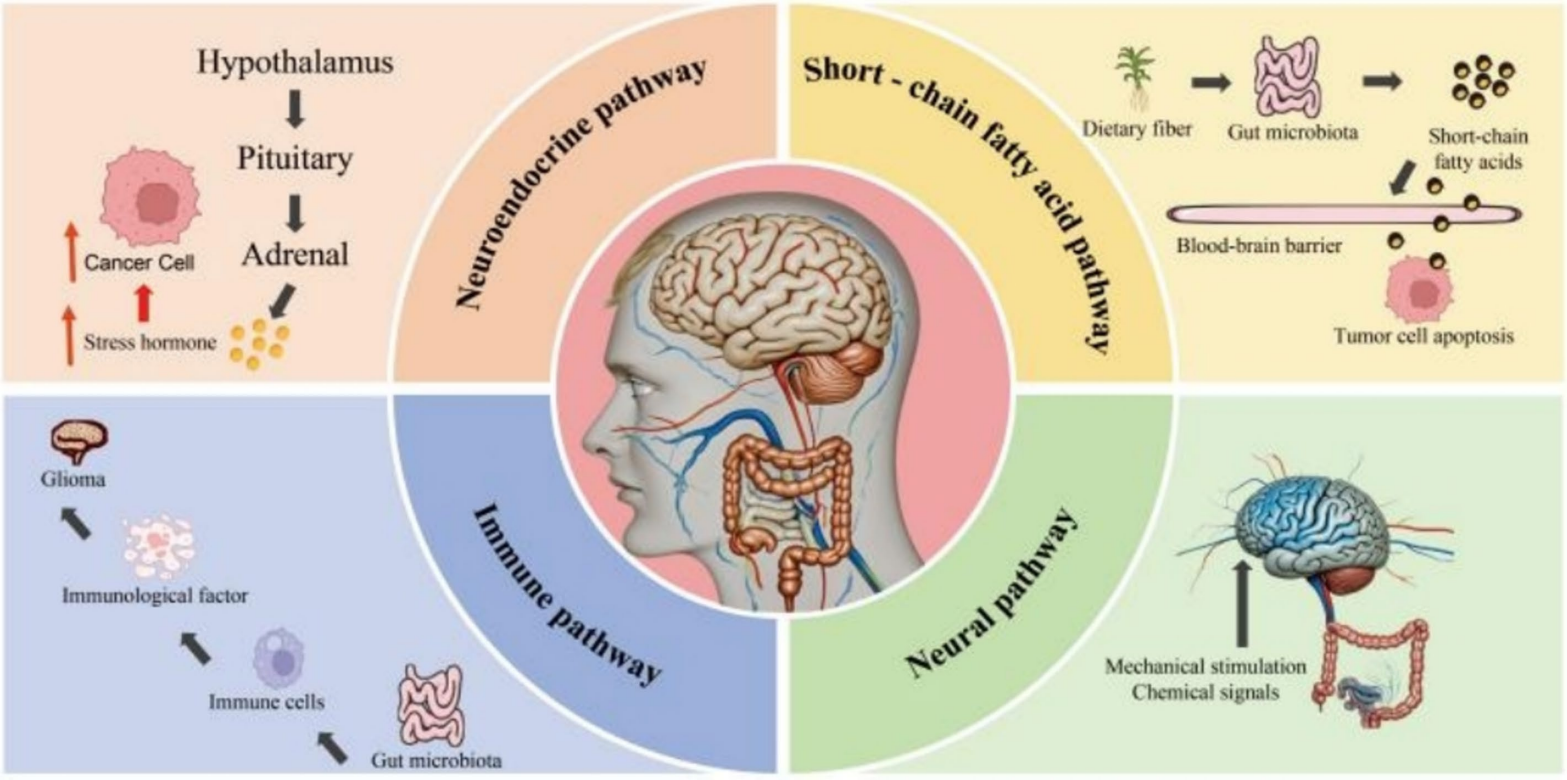
The key mechanisms of gut microbiota interfering with glioma through the gut-brain axis.

In the aspect of glioma, the influence pathways of the gut-brain axis mainly include the following four aspects. Firstly, the neuroendocrine pathway. Normal gut microbiota can maintain the development and integrity of the hypothalamus-pituitary-adrenal (HPA) axis. The gut microbiota can regulate the HPA axis to affect the secretion of stress hormones such as adrenocorticotropic hormone and serum cortisol, thus influencing the brain’s stress response and mood regulation (66). In glioma patients, gut microbiota dysbiosis can cause the overactivation of the HPA axis, leading to an increase in stress hormones, which suppresses the immune function of the body and further promotes the growth and metastasis of tumor cells (67). Secondly, the immune pathway. The intestine is the largest immune organ in the human body. The molecular structures related to gut microbiota can combine with intestinal epithelial cells and immune cells, releasing cytokines to affect the immune system and further influencing the inflammatory state of the brain and the function of the central nervous system (68, 69). For example, microbe-derived peptides, which are metabolites of the gut microbiota, enhance the immune system’s ability to recognize and attack glioblastoma by activating tumor-infiltrating lymphocytes, potentially inhibiting tumor progression (70, 71). Thirdly, the neural pathway. The vagus nerve is one of the most important neural pathways in the gut-brain axis. It transmits mechanical and chemical signals from the intestine to the brain and regulates the brain’s feedback to the intestine (72). If the gut microbiota is dysregulated, information will be transmitted to the brain through the vagus nerve, causing stress emotions such as anxiety and depression in humans and acting synergistically with the stress response of the HPA axis (73). Research shows that intestinal probiotics such as Bifidobacterium and Lactobacillus act on the brain-gut axis by producing the neurotransmitter gamma-aminobutyric acid. This action inhibits the levels of stress hormones and reduces the activity of the two pathways, namely the hypothalamic-pituitary-adrenal (HPA) axis and the sympathetic nervous system. Therefore, we propose a bold hypothesis that when the chronic stress state of the body is reversed, it will inhibit the progression of glioma (74, 75). Finally, the short-chain fatty acid pathway. The gut microbiota can produce short-chain fatty acids through the fermentation and metabolism of dietary fiber. They regulate the TIME, especially by promoting the polarization of M1-type macrophages with anti-tumor functions, thereby activating the body’s specific immune response and inhibiting the malignant progression of glioma (76). In conclusion, gut microbiota regulates the information communication between the gut and the brain through various action pathways, promotes the formation of the tumor inflammatory microenvironment, and provides stress pathways for the multi—factor environment of the glioma TME (Figure 2).

In conclusion, gut microbiota has a wide range of effects on the functions of the central nervous system under both physiological and diseased conditions. Under physiological conditions, gut microbiota participates in multiple key processes of the nervous system, including the formation and regulation of the blood–brain barrier, the formation of myelin sheaths, the generation of neurons, and the maturation and differentiation of microglia. Meanwhile, it also regulates the expression of neurotrophic factors and neurotransmitters. Under pathological conditions, gut microbiota can upregulate glioma through neuroendocrine, neuroimmune, and metabolic pathways, especially by regulating the maturation and activation of peripheral and central immune cells, thereby affecting the progression of glioma.

## 4 The impact of gut microbiota on the TME

An increasing amount of evidence on molecular mechanisms indicates that the interactions among gut microbiota, the brain, and the TME play an important role in the pathogenesis of glioma. In the TME of glioma, tumor cells interact with immune cells, stromal cells, and intratumoral microbiota, etc., releasing cytokines to target and regulate cancerous transcriptional programs. They also multidimensionally regulate the TME and the inflammatory microenvironment through systemic or local actions, thereby regulating the progression of glioma.

### 4.1 The impact of gut microbiota on the function of glioma cells

The metabolism of gut microbiota can produce products such as butyric acid and secondary bile acids. These small molecular compounds are absorbed into the blood by intestinal epithelial cells and can directly act on glioma cells through the blood–brain barrier via the blood circulation. Studies have found that butyric acid may have the effect of inhibiting the proliferation of glioma cells. Meanwhile, it can regulate cell metabolism, affect the cell cycle process, and induce apoptosis of glioma cells (77). Besides directly acting on glioma, secondary bile acids can also affect the metabolism of glioma cells through the gut-brain axis cycle. By regulating tumor signal pathways, they can reshape the TME and then regulate the migration and invasion abilities of glioma cells (78). Studies also show that the metabolism of gut microbiota can produce neurotransmitters such as serotonin and dopamine. In the glioma TME, serotonin can affect the activity and function of immune cells, thereby having an impact on the development of glioma. Meanwhile, it may participate in regulating the proliferation signal pathway of glioma cells, thus controlling the growth rate of tumor cells (79). Dopamine can regulate the energy supply and substance synthesis by affecting the metabolic pathways of glioma cells, thereby influencing the proliferation of glioma cells (80). Unfortunately, it is currently unclear whether the effects of the gut microbiota on serotonin and dopamine levels have significant biological or clinical significance against the backdrop of the high intrinsic concentrations of these substances in the brain. Further research is needed to determine this.

### 4.2 The mechanism of gut microbiota intervening in tumor-associated immune cells

The interactions between gut microbiota and the TME jointly maintain the balance of the body’s tumor state. The interactions and feedback mechanisms between gut microbiota and the host immune system together form a systemic microbiota-immune network. This complex interrelationship increases the possibility of regulating the tumor progression.

Through an experiment of introducing *Lactobacillus reuteri* into the intestines of germ-free mice, it was found that *Lactobacillus reuteri* could significantly increase the number of T cells in the mice, confirming that gut microbiota can regulate the inflammatory response in the intestines and maintain intestinal immune homeostasis. It can be seen that gut microbiota is closely related to maintaining the host’s immunity (81). The interaction between gut microbiota and the immune system helps to maintain the host’s immune balance. Studies have shown that gut microbiota can affect the differentiation and function of T cells and change the proportion of T cell subsets. For example, specific gut probiotics such as those of the Bifidobacterium genus and Lactobacillus genus can stimulate DCs to secrete specific cytokines, which then guide T cells to differentiate into Th1 cells, activate TAMs and enhance the activity of CTLs, jointly participating in anti-tumor immunity (82, 83). Other studies have also found that gut microbiota can enhance the activity of NK cells, promote the infiltration and activation of NK cells at the tumor site, stimulate them to secrete cytokines such as IFN-γ, and enhance their killing ability against tumor cells (84). Intestinal Bacteroides promotes the progression of glioma by regulating the tumor infiltration of CD8+ T cells and suppressing their anti-tumor functions (85). Imbalance of the gut microbiota can downregulate the signal transduction of granulocyte-macrophage colony-stimulating factor, which mediates an increase in reactive oxygen species. This, in turn, increases the number of myeloid-derived suppressor cells (MDSCs), suppresses the function of T cells, and ultimately accelerates the progression of glioma (86).

The metabolites of gut microbiota can enter the brain through the blood circulation, affect the TIME in the brain, and regulate the activity of immune cells. In the glioma TME, the metabolites of gut microbiota can regulate the polarization direction of TAMs, influence their anti-tumor or pro-tumor functions in the TME, change the efficacy of tumor immunotherapy, and thus affect the tumor development process (87). For example, indole can promote the polarization of TAMs toward the M1 type through the mTOR signaling pathway, secrete pro-inflammatory cytokines such as TNF-α and IL-12, promote the Th1 type of immune response, and enhance the killing ability against tumor cells. While kynurenine can promote the polarization of TAMs toward the M2 type by activating the aryl hydrocarbon receptor (AhR), secrete immunosuppressive cytokines such as IL-10 and TGF-β, and promote the growth and transfer of tumors (88, 89). In addition, gut microbiota dysbiosis can also reduce the expression of Foxp3 on glioma cells and affect the immune balance between anti-inflammatory Tregs and pro-inflammatory Th17 cells to thereby inhibiting the growth of tumor cells (90, 91). This regulation is significantly associated with the poor clinical outcomes of glioma, and the microbial-related transcripts of tumors can further explain the mechanism by which gut microbiota dysbiosis promotes the progression of glioma. In addition, the application of the gut microbiota in immune checkpoint blockade therapy offers new ideas for glioma treatment. This therapy promotes the migration of the gut microbiota to extra-intestinal tissues by reshaping lymph nodes and activating dendritic cells, thereby regulating the expression levels of PD-1 and CTLA-4 on the surfaces of glioma cells and immune cells. This extra-intestinal anti-tumor immune mechanism can not only enhance the overall anti-tumor ability of the body but also inhibit the growth of glioma cells and improve the survival rate of patients through a dual-action mechanism (92).

### 4.3 The impact of gut microbiota on tumor-related cytokines

Imbalance of the gut microbiota may disrupt the integrity of the intestinal barrier, leading to the abnormal infiltration of pro-inflammatory cytokines into the bloodstream (93). This chronic low-grade inflammatory microenvironment can activate tumor-related signaling pathways, promote the invasion and metastasis of glioma cells, and increase the expression of immune checkpoint molecules. Ultimately, this accelerates the malignant progression of glioblastoma (94).

The main metabolites of gut microbiota are short-chain fatty acids, and specific changes in short-chain fatty acids play an important regulatory role in the prevention and treatment of glioma. Studies have found that short-chain fatty acids can regulate the function of immune cells and affect the inflammatory response (95). In terms of glioma, short-chain fatty acids can inhibit the proliferation of tumor cells and induce apoptosis of glioma cells. Short-chain fatty acids can also reduce the expression levels of IL-10 and TGF-β, and then improve the glioma TIME by promoting the proliferation of lymphocytes and the differentiation of T cells, as well as increasing the proportion of M1-type TAMs in the TME, thereby improving the prognosis of glioma patients (76). Secondly, the non-short-chain fatty acids produced by the metabolism of gut microbiota can affect the regulation of astrocytes on nerve excitability and synapse formation, thus limiting the occurrence of T cell-dependent inflammation in the central nervous system and inhibiting the invasion of glioma cells (96, 97).

The metabolites of gut microbiota may also indirectly affect the activation state of cancer-associated fibroblasts CAFs by regulating the cytokines secreted by immune cells. The over-activated CAFs secrete pro-tumor growth factors such as vascular endothelial growth factor VEGF and PDGF as well as extracellular matrix components, which promote the growth and invasion of glioma. In addition, gut microbiota dysbiosis may lead to abnormal functions of vascular endothelial cells and pericytes. Under the influence of various pro-tumor cytokines, it further promotes the angiogenesis and leakage of tumor blood vessels, providing more nutrients and oxygen for the growth of glioma (98, 99).

### 4.4 The impact of gut microbiota on intratumoral microbiota and other aspects

Existing research indicates that there seems to be a close correlation between the gut microbiota and glioma. The gut microbiota may directly affect glioma through the gut-brain axis. In addition, the gut microbiota interacts with various components in the TME, thus indirectly influencing the growth environment of glioma. Although there are currently few research findings regarding the intratumoral microbiota in glioma, as an important part of the tumor microenvironment, the intratumoral microbiota must play a crucial role in the development of glioma. In subsequent research, identifying treatment targets related to the intratumoral microbiota is expected to become a new direction for future glioma treatment.

In addition, the gut microbiota also has a definite impact on the orally administered therapeutic drugs for glioma, which in turn affects the phenotype of glioma cell (100). Other studies have also found that the abundance of gut bacteria and related metabolites is closely related to the pharmacodynamic indicators of temozolomide. Temozolomide can enhance the metabolism of Akkermansia and Bifidobacterium in the body, thus affecting the efficacy of chemotherapy drugs and the occurrence of side effects (101, 102).

In conclusion, gut microbiota dysbiosis is the main cause of the formation of hypoxic, acidic, inflammatory and even interstitial hypertensive environments. It promotes the formation of the TME and provides favorable conditions for the generation, development and metastasis of glioma. On the one hand, it can directly affect the growth and metastasis of glioma cells through metabolites and signal molecules. On the other hand, through the interaction with the host immune system, gut microbiota can indirectly regulate the function and distribution of immune cells, thereby affecting the tumor’s immune escape and anti-tumor immune response. Therefore, targeting the regulation of gut microbiota to improve the TME is expected to become a new strategy for the treatment of glioma in the future.

## 5 Conclusion and prospect

In glioma, the relationship between gut microbiota and the TME mainly involves gut microbiota dysbiosis, inflammation-related damage, and tumor immune resistance. The TME promotes the development of glioma by regulating the tumor’s nutrient supply, growth, immune escape, and invasion and metastasis. The changes in gut microbiota will not only directly affect the proliferation of tumor cells but also further influence the formation of the tumor inflammatory microenvironment and TIME by affecting the secretion of cytokines and neurotransmitters, as well as the changes in metabolites and immune functions, thus regulating the growth of glioma. Overall, the gut microbiota not only acts on the central nervous system through the bidirectional regulation mechanism of the gut-brain axis, by secreting metabolites and mediating intercellular signal transduction. At the same time, as a key regulator of the TME, it drives the malignant progression of glioma by reshaping the immunosuppressive characteristics and metabolic homeostasis of the TME, thus playing a dual-regulatory role in tumorigenesis, development, and clinical outcomes.

Despite the progress made in understanding the relationship among gut microbiota, the TME, and glioma, there are still certain limitations and areas that require more in-depth research. The exact mechanisms by which gut microbiota dysbiosis leads to changes in the TME and glioma have not been fully elucidated. Further research is needed to determine the specific molecular mechanisms and signal pathways involved in this relationship. Currently, there is a lack of reliable biomarkers to assess the changes in gut microbiota and the TME in glioma patients. Developing specific markers that can accurately measure the changes in microbial communities is crucial for the early diagnosis of glioma patients and monitoring the progression of glioma. In addition, developing animal models that are closer to human diseases will further promote research in the field of glioma. It is necessary to conduct clinical trials on these pathways in glioma patients to evaluate their efficacy. Therefore, we look forward to exploring more precise and effective methods from the perspectives of gut microbiota and the TME in the future diagnosis and treatment of glioma.
